# Activation of mGluR2/3 underlies the effects of N-acetylcystein on amygdala-associated autism-like phenotypes in a valproate-induced rat model of autism

**DOI:** 10.3389/fnbeh.2014.00219

**Published:** 2014-06-17

**Authors:** Yu-Wen Chen, Hui-Ching Lin, Ming-Chong Ng, Ya-Hsin Hsiao, Chao-Chuan Wang, Po-Wu Gean, Po See Chen

**Affiliations:** ^1^Department of Pharmacology, College of Medicine, National Cheng-Kung UniversityTainan, Taiwan; ^2^Department and Institute of Physiology, School of Medicine, National Yang-Ming UniversityTaipei, Taiwan; ^3^Brain Research Center, National Yang-Ming UniversityTaipei, Taiwan; ^4^Department of Psychiatry, College of Medicine, National Cheng Kung University Hospital, National Cheng Kung UniversityTainan, Taiwan; ^5^Department of Anatomy, College of Medicine, Kaohsiung Medical UniversityKaohsiung, Taiwan; ^6^Addiction Research Center, National Cheng Kung UniversityTainan, Taiwan

**Keywords:** amygdala, autistic spectrum disorders, valproate, *N*-acetylcysteine, glutamate

## Abstract

Autism-like phenotypes in male valproate (VPA)-exposed offspring have been linked to high glutamatergic neurotransmission in the thalamic-amygdala pathway. Glial cystine/glutamate exchange (system Xc^−^), which exchanges extracellular cystine for intracellular glutamate, plays a significant role in the maintenance of extracellular glutamate. *N*-acetylcysteine (NAC) is a cystine prodrug that restores extracellular glutamate by stimulating system Xc^−^. In this study, we examined the effects of NAC on autism-like phenotypes and neurotransmission in the thalamic–amygdala synapses, as well as the involvement of metabotropic glutamate receptors 2/3 (mGluR2/3). Valproate-treated rats received a single intraperitoneal injection of 500 mg/kg NaVPA on E12.5. On postnatal day 21 (P21), NAC or saline was administered once daily for 10 days. From day 8 to 10, NAC was given 1/2 h prior to behavioral testing. Chronic administration of NAC restored the duration and frequency of social interaction and ameliorated anxiety-like behaviors in VPA-exposed offspring. In amygdala slices, NAC treatment normalized the increased frequency of mEPSCs and decreased the paired pulse facilitation (PPF) induced by VPA exposure. The effects of NAC on social interaction and anxiety-like behavior in the VPA-exposed offspring were blocked after intra-amygdala infusion of mGluR2/3 antagonist LY341495. The expressions of mGluR2/3 protein and mGluR2 mRNA were significantly lower in the VPA-exposed offspring. In contrast, the mGluR3 mRNA level did not differ between the saline- and VPA-exposed offspring. These results provide the first evidence that the disruption of social interaction and enhanced presynaptic excitatory transmission in VPA-exposed offspring could be rescued by NAC, which depends on the activation of mGluR2/3.

## Introduction

Autism spectrum disorders (ASDs) are a group of clinically and genetically heterogeneous neurodevelopmental disorders characterized by social deficits, communication difficulties, stereotyped, or repetitive behaviors and interests (Baird et al., 2006; Levitt and Campbell, 2009). Several brain structures and pathways have been suggested to underlie this disorder, including the amygdala (Adolphs et al., 2001; Baron-Cohen and Belmonte, 2005). Functional magnetic resonance imaging (fMRI) study has provided evidence of abnormality of the amygdala in autism (Baron-Cohen et al., 1999, 2000). Altered amygdala activation in response to facial and emotion processing has been noted in individuals with ASD (Wang et al., 2004; Dalton et al., 2005; Hadjikhani et al., 2007). Postmortem studies of individuals with ASD have shown cyto-architectural and neuronal organization changes within the amygdala (Bailey et al., 1998; Schumann and Amaral, 2006). Structural MR imaging studies have also demonstrated abnormal amygdala volumes both in adolescents and adults with ASD (Aylward et al., 1999; Rojas et al., 2004; Nacewicz et al., 2006). These results suggest that amygdala dysfunction may contribute to core social impairment in ASD (Todd and Anderson, 2009).

Exposures to environmental toxicants that activate the immune system have been implicated in ASD (Windham et al., 2006; Roberts et al., 2007; Palmer et al., 2009). Rats with gestational exposure to lipopolysaccharide, an endotoxin that activates the immune system, have been established as a prenatal infection model for developmental neuropsychiatric disorders (Baharnoori et al., 2012). Intra-cerebroventricular injection of propionic acid, a short chain fatty acid, has also been used to develop a rodent model of ASD (Macfabe et al., 2007; Shultz et al., 2009; El-Ansary et al., 2012; Foley et al., 2014). In clinical practice, valproate (VPA), a short chain fatty acid, has been widely used for the treatment of patients with epilepsy and mood disorder. However, the offspring of women taking VPA medication during early pregnancy are at increased risk of ASD (Christensen et al., 2013; Wood, in press). In a rat model of ASD, VPA-exposure offspring exhibited several symptoms common to ASD and enhanced fear learning memories (Markram et al., 2008).

The precise mechanisms by which VPA-exposure offspring exhibit autism-like phenotypes are still not clear. In VPA-exposed offspring, Tyzio et al. observed increased excitatory GABA, glutamatergic activity and gamma oscillations in hippocampal CA3 neurons (Tyzio et al., 2014). Importantly, restoration of the neuronal excitatory and inhibitory balance could rescue the behavioral phenotypes. We have also observed enhanced long term potentiation at the thalamic–amygdala synapses in VPA-exposed offspring (Lin et al., 2013). Furthermore, treatment with a 5-HT1A receptor agonist increased social interaction and also reversed the characteristics of miniature excitatory post-synaptic currents as well as paired pulse facilitation (PPF) observed in lateral amygdala slices (Wang et al., 2013). A functional balance between neuronal excitatory and inhibitory systems is established during development (Eichler and Meier, 2008). Defects in the ability to establish and maintain the balance have been considered to play a key role in the pathogenesis of ASD (Bateup et al., 2013). In a genetic rodent model of ASD, autistic-like behaviors are also accompanied by an increased ratio of excitatory to inhibitory synaptic inputs in the medial prefrontal cortex, striatum, and hippocampus (Gkogkas et al., 2013; Santini et al., 2013). These studies indicated that excitatory/ inhibitory imbalances in key brain regions could be associated with the behavioral phenotypes of ASD.

N-acetylcysteine (NAC) is an antioxidant and a precursor of glutathione that has been shown to rescue the brain from free radical injury after focal cerebral ischemia (Khan et al., 2004). NAC also reversed cognitive impairment and oxidative stress in aged and Tg2576 mutant mice (Farr et al., 2003; Fu et al., 2006; Parachikova et al., 2010). The antiporter system Xc^−^, which belongs to the family of heterodimeric amino acid transporters, imports the amino acid cystine into cells with a 1:1 counter-transport of glutamate. NAC is an orally bioavailable prodrug of cysteine that can be oxidized to cystine. After administration of NAC, Xc^−^ antiporter allows for the cellular uptake of cystine, which causes the reverse transport of glutamate into the extracellular space. The nonvesicular glutamate released into the extracellular space stimulates the type 2/3 metabotropic glutamate receptors (mGluR2/3), which, in turn, inhibit the vesicular release of glutamate, thereby resulting in a decrease in glutamatergic neurotransmission (Baker et al., 2002). In clinical trials, administration of NAC or a placebo in a blind fashion to a group of children with autistic disorder supports the potential usefulness of NAC for treating behavioral disturbance in children with autism (Hardan et al., 2012). The purpose of this study was to determine whether NAC normalizes the increased presynaptic transmitter release and decreased social interaction seen in VPA-exposed offspring. We demonstrated that NAC reversed abnormal behaviors and synaptic transmission in a VPA-induced rat ASD model by acting on the mGluR2/3 receptors.

## Materials and methods

### Animals

We used the valproate-induced rat model of autism. All procedures were approved by the Institutional Animal Care and Use Committee of the College of Medicine, National Cheng-Kung University (Tainan, Taiwan). Sprague Dawley rats were housed in groups of 3–4 per cage in a temperature-controlled (24°C) vivarium on a 12 h reverse light/dark cycle, with lights on at 7:00 AM. All behavioral procedures took place during the light cycle. Rats were mated, with pregnancy determined by the presence of a vaginal plug on embryonic day 1 (E1). The sodium salt of valproic acid (NaVPA, Sigma-Aldrich, MO, USA) was dissolved in 0.9% saline to obtain a concentration of 150 mg/mL, pH 7.3. Exposed dams received a single intraperitoneal injection of 500 mg/kg NaVPA adjusted according to body weight and control dams a single injection of saline on E12.5. Dams were housed individually and were allowed to raise their own litters until weaning. The offspring were then separated and housed in cages of 3–4 rats until the end of all behavioral experiments.

### Behavioral testing

**Figure 1:**
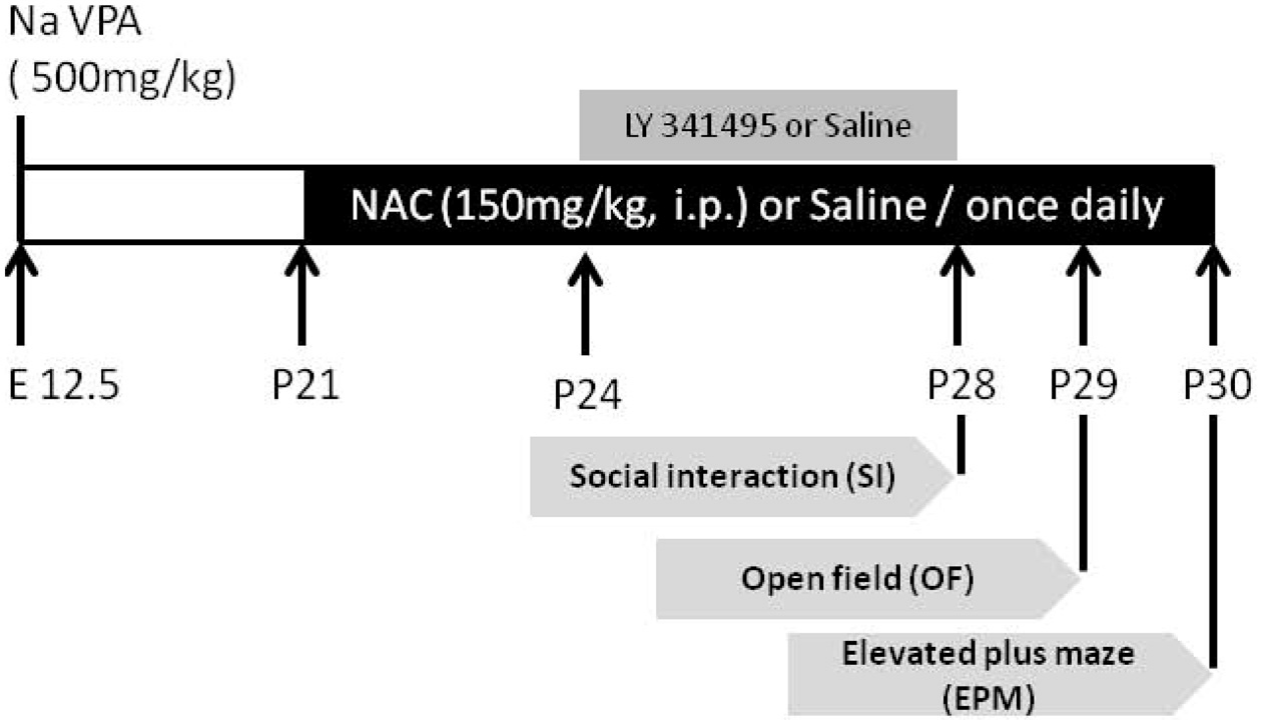
**The experimental design employed in the present study**. On postnatal day 21 (P21), NAC (150 mg/kg, intraperitoneal (i.p.); sigma) or saline was administered once daily for 10 days. From P28 to P30, the social interaction test, elevated plus-maze test, and open field test were conducted each day after NAC treatment. To elucidate whether the effect of NAC on social interaction in VPA-exposed offspring is mediated by mGluR2/3, on P24, the mGluR2/3 antagonist LY341495 (2 μg/0.5 μl/side) was infused into the amygdala 30 min before the administration of NAC once per day for 5 days.

On postnatal day 21 (P21), NAC (150 mg/kg, intraperitoneal (i.p.); sigma) or saline was administered once daily for 10 days. From day 8 to 10, NAC was given 1/2 h prior to behavioral testing (Figure 1). The behavioral tests were analyzed in the saline-exposed with vehicle (saline/saline), saline-exposed with NAC (saline/NAC), VPA-exposed with vehicle (VPA/saline), or VPA-exposed with NAC (VPA/NAC) offspring. The social interaction test, elevated plus-maze test and open field test were conducted each day after NAC treatment. At the end of the elevated plus-maze test, the same offspring received either electrophysiological recordings or were sacrificed for western blotting. Video tracking software (Ethovision, Noldus, Netherlands) was used for automatic recording and analysis of social interaction.

#### Social interaction

Rats were separated and housed individually the night before the experiment. The apparatus was a white plastic box (50 × 40 × 40 cm). Rats were matched in terms of age, gender, and weight. After a 1-h habituation period in the room, one VPA-exposed and one saline-exposed rat were placed into the apparatus over a period of 20 min. The percentage of time spent sniffing of any body part, mounting, grooming each other, and following were taken as indicators of social engagement (Flagstad et al., 2004).

#### Open field test

The rats were placed in a white plastic box (50 × 40 × 40 cm) for 15 min. The percentage of time spent in the central zone (25% of the surface area) and the total distance (cm) moved were measured.

#### Elevated plus-maze

The rats were placed in a standard elevated plus-maze for 5 min, which consisted of a plus-shaped apparatus with two opposite open arms and two opposite enclosed arms (50 × 5 × 40 cm) arranged at right angles. The percentage of time spent in each arm and the total distance (cm) moved were measured.

### Brain slice preparation and electrophysiological recordings of the amygdala

Brain slices were prepared as described previously (Lin et al., 2013). Whole-cell recordings were made from the soma of visually-identified pyramidal-like neurons located in the LA. Neurons were identified as projection neurons based on their intrinsic electrophysiological properties (Washburn and Moises, 1992) in potassium gluconate-containing electrodes. The miniature excitatory post-synaptic currents (mEPSCs) were recorded in the presence of bicuculline (10 μM; Tocris) and tetrodotoxin (TTX, 5 M; Tocris). We also examined the PPF in slices from the saline/saline, saline/NAC, VPA/saline, and VPA/NAC offspring. The ratio of the amplitude of the first EPSC divided by the amplitude of the second EPSC was examined at 30, 50, 150, and 200-ms interpulse intervals.

### Surgery

Both saline-exposed and VPA-exposed offspring were anesthetized with 10% chloral hydrate (50 mg/kg, i.p.). The mice were mounted on a stereotaxic apparatus and cannulas (22 gauge stainless steel tubing) were implanted into the lateral amygdala (LA) (anteroposterior, −2.3 mm; mediolateral, ±5 mm; dorsoventral, −6.7 mm). A 28-gauge dummy cannula was inserted into each cannula to prevent clogging. The rats were monitored and handled daily and were given 7 days to recover. The mGlu2/3 antagonist LY341495 (Tocris), dissolved in about 10 μl of 0.1 M NaOH and brought to dose volume using 0.9% sterile saline solution and adjusted to pH 7.4, was administered bilaterally to the amygdala in a volume of 2 μg/0.5 μl per side at a rate of 0.1 μl/min 30 min before the last NAC administration and each behavioral test. The infusion cannulas were left in place for 2 min before being withdrawn.

### Western blotting assay

After all behavioral tests, amygdala tissues were dissected from the brains of rats of both groups. The amygdala tissues were lysed in a lysis buffer containing 1% Triton X-100, 0.1% SDS, 50 mM Tris-HCl, pH 7.5, 0.3 M sucrose, 5 mM EDTA, 2 mM sodium pyrophosphate, 1 mM sodium orthovanadate, and 1 mM enylmethylsulfonyl fluoride, supplemented with complete protease inhibitor cocktail. Following sonication, the lysates were centrifuged at 12,000 rpm for 30 min to obtain supernatants. The protein concentrations of the supernatants were measured using a Bradford assay, and equal amounts of protein were separated by SDS-PAGE electrophoresis, transferred to Immobilon-P membranes (Millipore), and incubated in 5% nonfat dry milk for 60 min. Western blot analysis involved mGluR2/3 (1:1000; Abcam), Xc^−^ (1:1000; Abcam) and Actin (1:100000; Millipor) antibody reacted overnight at 4°C and incubated with HRP-conjugated secondary antibodies for 1 h at room temperature. Immunoreactivity was detected by ECL Plus detection reagent (PerkinElmer, Boston, MA). Films were exposed at different times to ensure optimum density but not saturation, followed by densitometry. Protein levels were first normalized to internal control levels for each sample and then measured as fold changes of those in the controls, respectively. Three replicates were performed for each experiment.

### *Grm2* and *Grm3* mRNA measurement

A total RNA spin kit (Bioman) was used to extract total RNA from amygdala tissues and the ImProm-II Reverse Transcription System (Promega Madison, WT) was used to synthesize cDNA. mRNAs of *Grm2* and *Grm3* were measured by TaqMan real-time quantitative reverse transcription polymerase chain reaction (RT-PCR) using a StepOnePlus™ System (Life Technologies). A Grm2 probe (Rn01447672_m1), Grm3 probe (Rn01755352_m1), and glyceraldehyde-3-phosphate dehydrogenase (GAPDH) probe (Rn01775763_g1) were obtained from Life Technologies. The RT reactions were performed as follows: 50°C for 2 min, 95°C for 10 min, followed by 40 cycles at 95°C for 15 s and 1 min at 60°C. The *Gapdh* gene was used as an endogenous control to standardize the amount of sample RNA. The levels of *Grm2* and *Grm3* mRNA were first normalized to *Gapdh* mRNA for each sample and then measured as fold changes of those in the controls.

### Statistical analysis

All values are given as mean ± s.e.m. Two-way ANOVA and Newman–Keuls *post-hoc* comparisons were used to analyze the differences in behavioral tests, electrophysiology responses and protein levels of saline-exposed and VPA-exposed rats. The variables were assessed using Student's *t*-test to analyze the differences in mGlur2/3 antagonist effects and mRNA level among the controls and the VPA-exposed group. The difference between groups was considered significant if *p* < 0.05.

## Results

### Effects of NAC on behavioral phenotypes of VPA-exposed offspring

**Figure 2:**
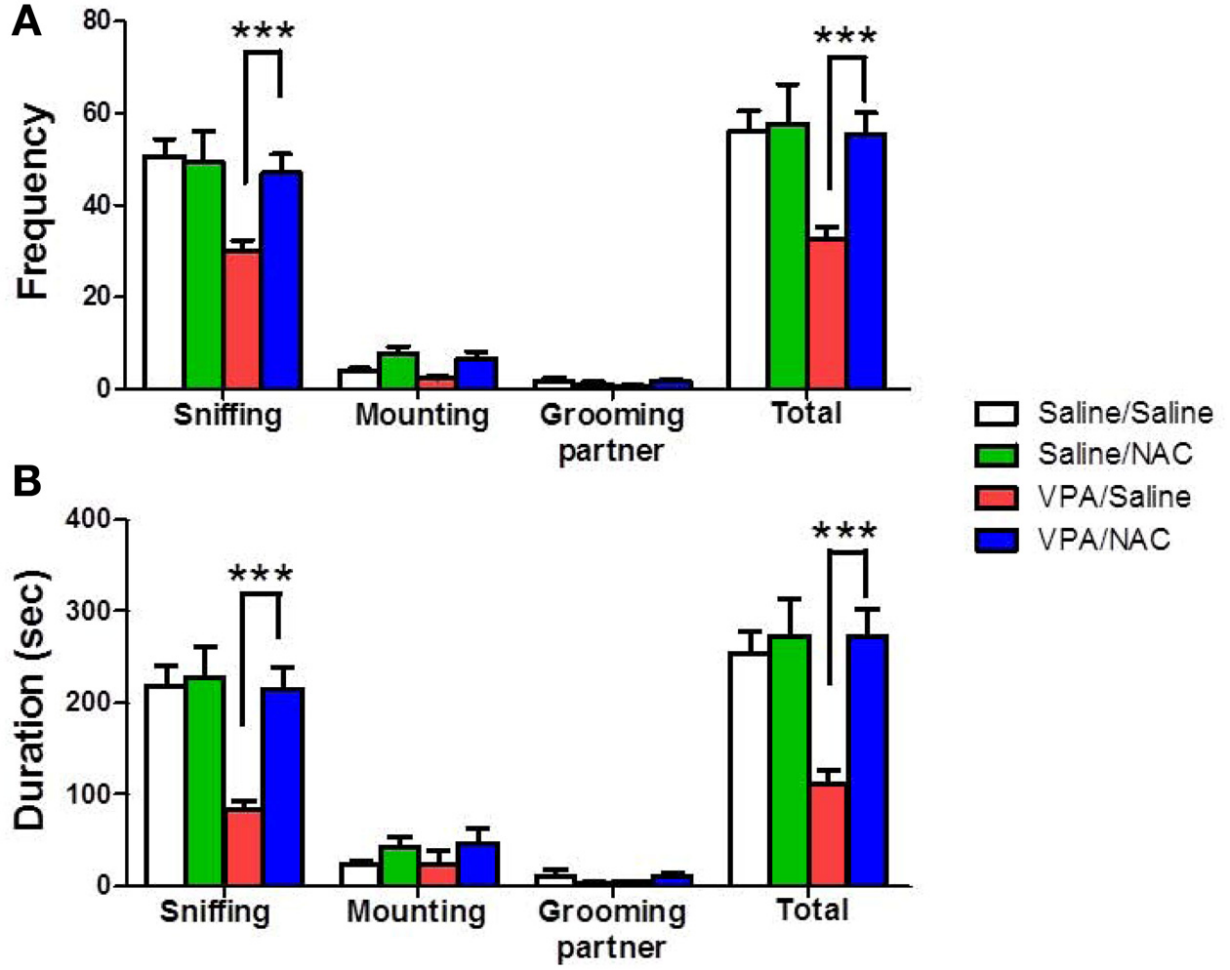
**Reversal of the social interaction deficit in VPA-exposed offspring by NAC**. Rats of VPA- or saline-expose offspring weaned at postnatal day 21 (P21) were used in this experiment. NAC (150 mg/kg, i.p.) or saline was administered once per day for 10 days (P21 to P30). On P28, the male offspring were tested for social interaction. The duration **(A)** and the frequency **(B)** of occurrence of various social behaviors such as sniffing, mounting and grooming partner were measured in the VPA- and saline-exposed offspring for 20 min. Sample sizes (*n*): Saline/Saline *n* = 21, Saline/NAC *n* = 5, VPA/Saline *n* = 25, VPA/NAC *n* = 17. ^***^p < 0.001 vs. VPA/NAC.

The rats were classified into saline/saline, saline/NAC, VPA/saline, and VPA/NAC 4 groups. We first determined whether NAC had any effect on social interaction deficit in male VPA-exposed offspring. The dose of NAC chosen was based on a previous report in which NAC (150 mg/kg) administered subcutaneously once daily for 4 weeks improved the cognition of 12-month-old SAMP8 mice (Farr et al., 2003). As shown in Figure 2A, male VPA-exposed offspring exhibited a lower duration of social interaction than male saline-exposed offspring (*p* < 0.001). Importantly, the deficit in social interaction was completely reversed after treatment with NAC. Two-Way ANOVA revealed main effects of pre-treatment [VPA vs. saline, *F*_(1, 73)_ = 7.209, *p* < 0.01] and drug [NAC vs. saline, *F*_(1, 73)_ = 7.333, *p* < 0.01] and a significant pre-treatment by drug interaction [*F*_(1, 73)_ = 4.175, *p* < 0.05]. A similar result was observed for the frequency of interaction (Figure 2B). Two-Way ANOVA revealed main effects of pre-treatment [VPA vs. saline, *F*_(1, 73)_ = 9.073, *p* < 0.01] and drug [NAC vs. saline, *F*_(1, 73)_ = 5.527, *p* < 0.05] and a significant pre-treatment by drug interaction [*F*_(1, 73)_ = 4.181, *p* < 0.05]. There were no differences in either the interaction duration or frequency between the saline/NAC and saline/saline groups. The results indicated that chronic treatment with NAC could ameliorate the social interaction deficits in the VPA-exposed offspring without affecting the performance of the saline-exposed offspring.

**Figure 3:**
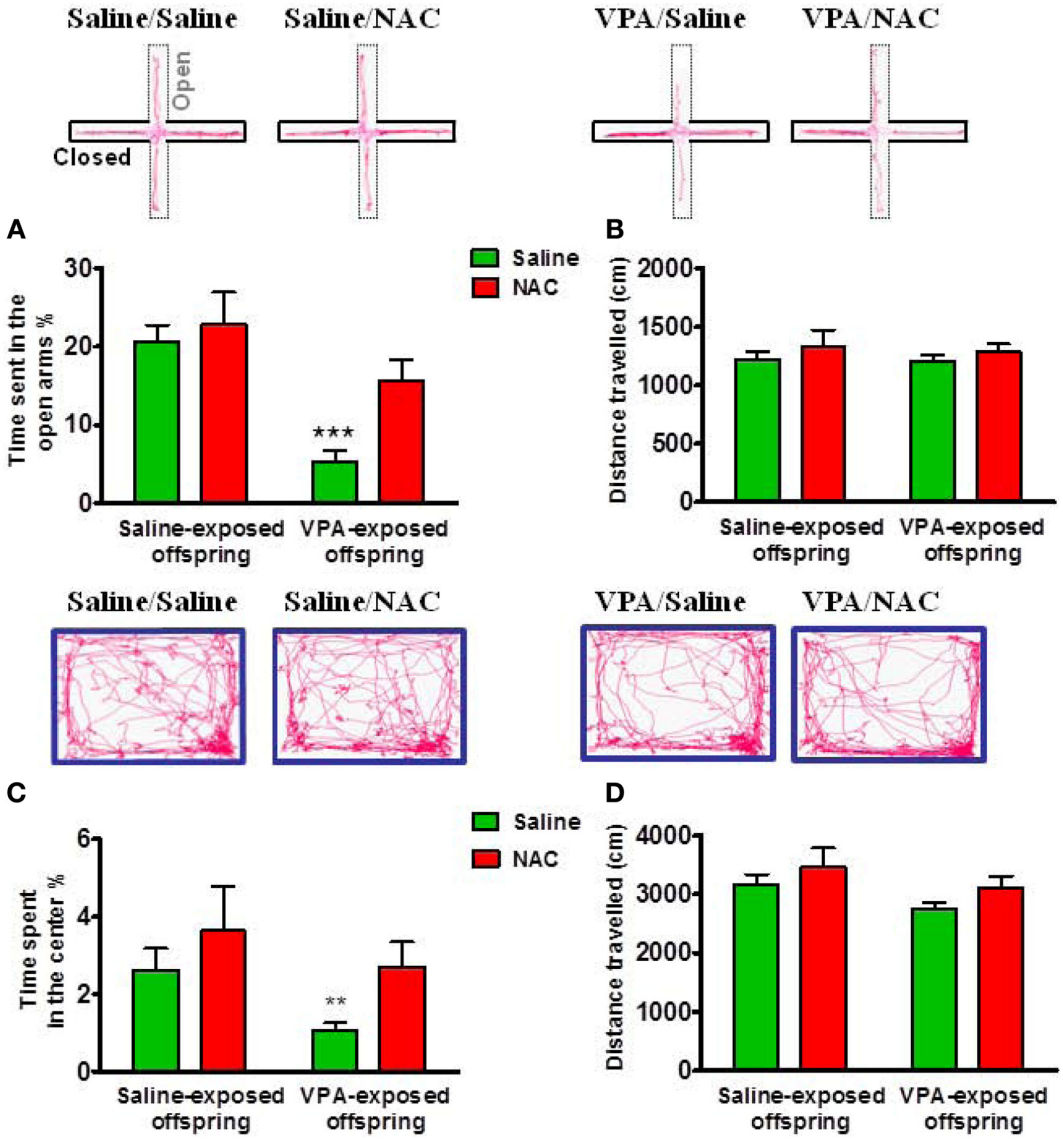
**Amelioration of anxiety in the VPA-exposed offspring by NAC**. On P29 and P30, rats were administered the elevated plus-maze test (EPM) and the open field test (OF). In the EPM test, the time spent in the open arms **(A)** and the distance traveled **(B)** were measured for 5 min. ^***^p < 0.001 vs. VPA/NAC. In the OF test, the time spent in the center **(C)** and the distance traveled **(D)** were measured for 15 min. ^**^p < 0.01 vs VPA/NAC.

To examine whether the VPA-exposed offspring exhibited emotional alterations, the rats were tested with anxiety-like behavioral paradigms. In the EPM test, Two-Way ANOVA revealed main effects of pre-treatment [VPA vs. saline, *F*_(1, 73)_ = 15.27, *p* < 0.001] and drug [NAC vs. saline, *F*_(1, 73)_ = 4.715, *p* < 0.05] but no significant pre-treatment by drug interaction [*F*_(1, 73)_ = 1.872, *p* > 0.05] (Figure 3A). VPA-exposed offspring exhibited less time spent in the open arms than saline-exposed offspring (*p* < 0.001). NAC treatment restored the time spent in the open arms. There was no difference in the distance traveled (Figure 3B). In the open field test, Two-Way ANOVA revealed main effects of pre-treatment [VPA vs. saline, *F*_(1, 64)_ = 4.053, *p* < 0.05] and drug [NAC vs. saline, *F*_(1, 64)_ = 4.578, *p* < 0.05] but no significant pre-treatment by drug interaction [*F*_(1, 64)_ = 0.27, *p* > 0.5] (Figure 3C). The VPA-exposed offspring exhibited less time spent in the center (*p* < 0.01), which was restored after treatment with NAC. There was no difference in the distance traveled (Figure 3D). These results suggest that VPA-exposed offspring exhibit anxiety-related behaviors that could be ameliorated by NAC.

### NAC modulates amygdala synaptic activity in VPA-exposed offspring

**Figure 4:**
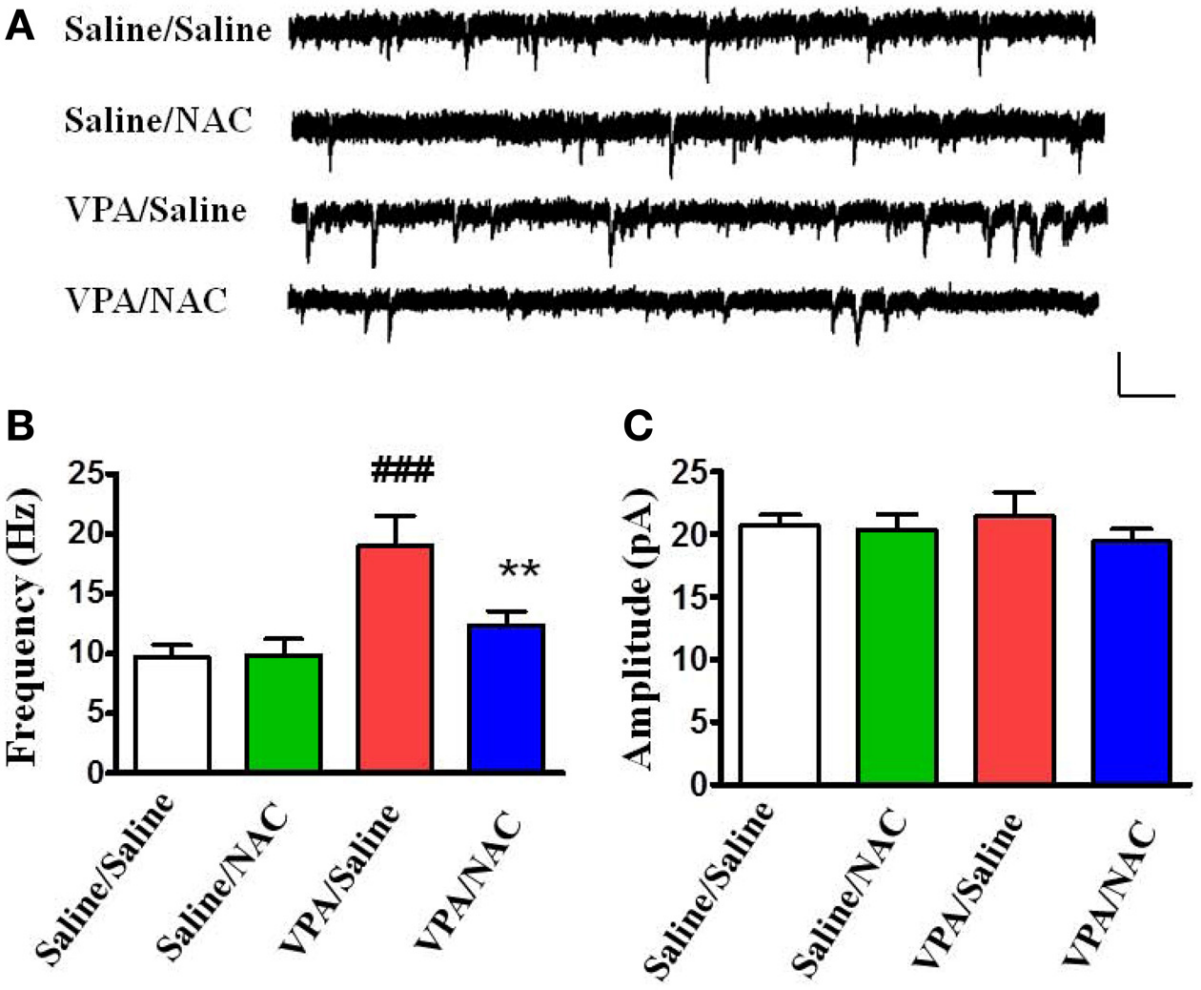
**Effects of NAC on the amplitude and frequency of mEPSCs recorded in the LA of VPA-exposed offspring. (A)** Sample traces of mEPSCs taken from slices of Saline/Saline, Saline/NAC, VPA/Saline, and VPA/NAC rats. mEPSCs were recorded in the LA neurons at a holding potential of −70 mV in the presence of TTX (1 μM). Calibration: 30 pA, 100 ms. **(B,C)** Summary plots of the frequency **(B)** and amplitude **(C)** of mEPSCs in the Saline/Saline, Saline/NAC, VPA/Saline, and VPA/NAC rats. ^**^p < 0.01 vs. VPA/saline. ^###^*p* < 0.001 vs. Saline/Saline.

We examined whether alteration of excitatory synaptic transmission in the VPA-exposed offspring could be normalized by NAC. Amygdala slices were made 1 h after the behavioral tests and whole-cell recordings were made from the soma of visually identified pyramidal-like neurons located in the LA. Figure 4 shows that saline/saline rats exhibited a significantly lower frequency of mEPSCs than the VPA/saline group (*p* < 0.001). Two-Way ANOVA revealed a significant main effect of pre-treatment [VPA vs. saline, *F*_(1, 24)_ = 25.35, *p* < 0.01] in the absence of a main effect of drug [NAC vs. saline, *F*_(1, 24)_ = 7.64, *p* = 0.0815] or a significant pre-treatment x drug interaction [*F*_(1, 24)_ = 8.36, *p* = 0.0698]. *Post-hoc* tests showed that the increased mEPSC frequency in the VPA-exposed offspring was significantly reduced after treatment with NAC (*p* < 0.01) (Figure 4B). The amplitudes of mEPSCs did not differ among the four groups (Figure 4C).

**Figure 5:**
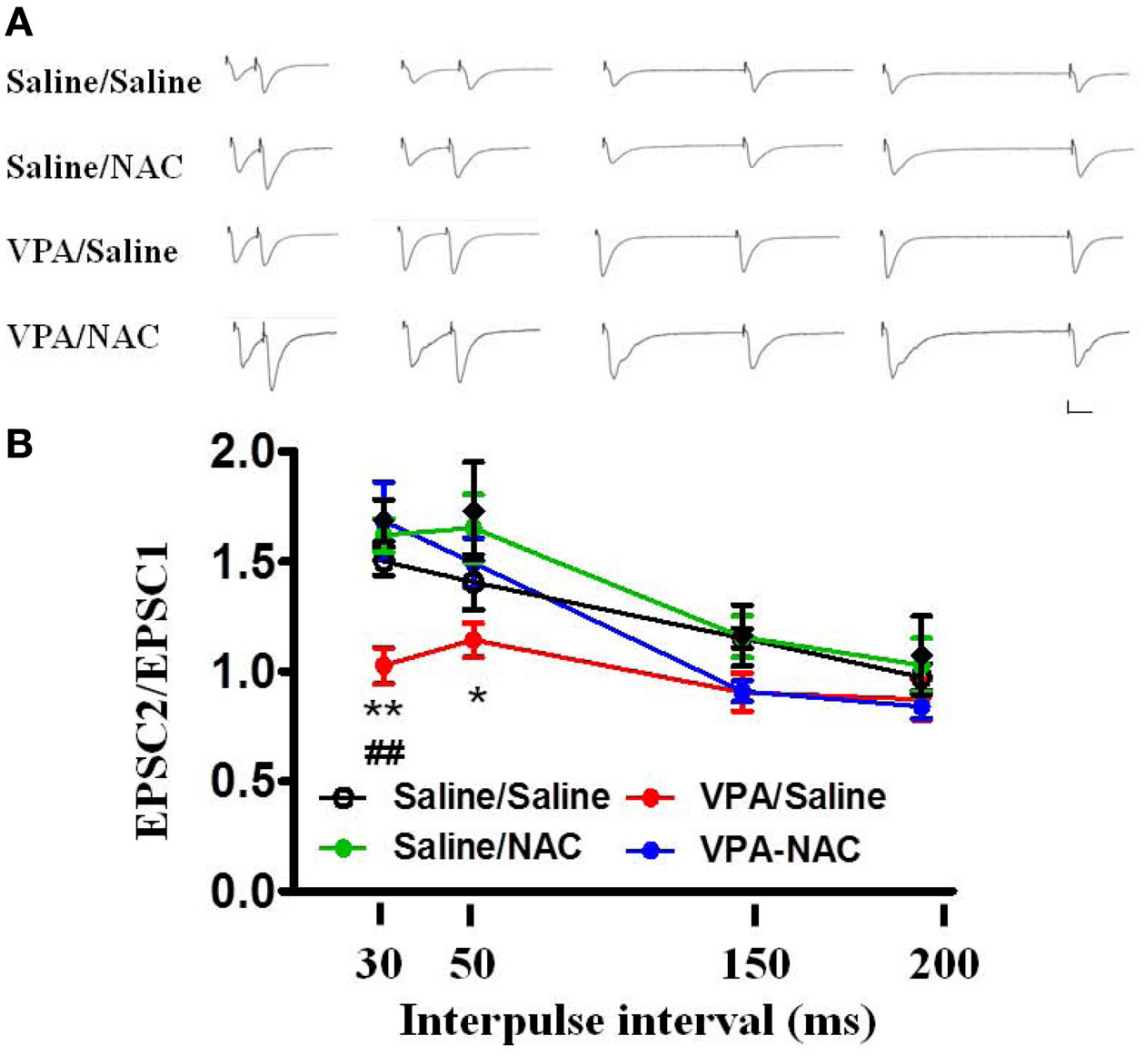
**Effects of NAC on the PPF of EPSCs at the thalamo-LA synapses of the VPA-exposed offspring. (A)** Sample traces of the PPF of EPSCs taken from slices of Saline/Saline, Saline/NAC, VPA/Saline, and VPA/NAC rats. Sample traces were the average of 3–5 successive responses. Calibration; 50 pA, 30 ms. **(B)** Plot of the PPF in the Saline/Saline, Saline/NAC, VPA/Saline, and VPA/NAC rats. ^*^p < 0.05, ^**^p < 0.01 vs. VPA/saline. ^##^*p* < 0.01 vs. Saline/Saline.

We further analyzed the PPF in the four groups. As illustrated in Figure 5, the PPF ratios at an interpulse interval of 30 ms were significantly lower in the VPA/saline group (1.03 ± 0.08, *n* = 8) than in the saline/saline (1.50 ± 0.06, *n* = 6, *p* < 0.01) rats. Administration of NAC restored the PPF ratio to a level comparable to that of the saline/saline group (1.68 ± 0.18, *n* = 8, *p* < 0.01 vs. VPA/saline). Moreover, there was no difference in the PPF between the saline/NAC and saline/saline groups. The results suggested that NAC normalizes the increased presynaptic transmitter release in the VPA-exposed offspring.

### Effect of NAC on social interaction in VPA-exposed offspring is mediated by mGluR2/3

**Figure 6:**
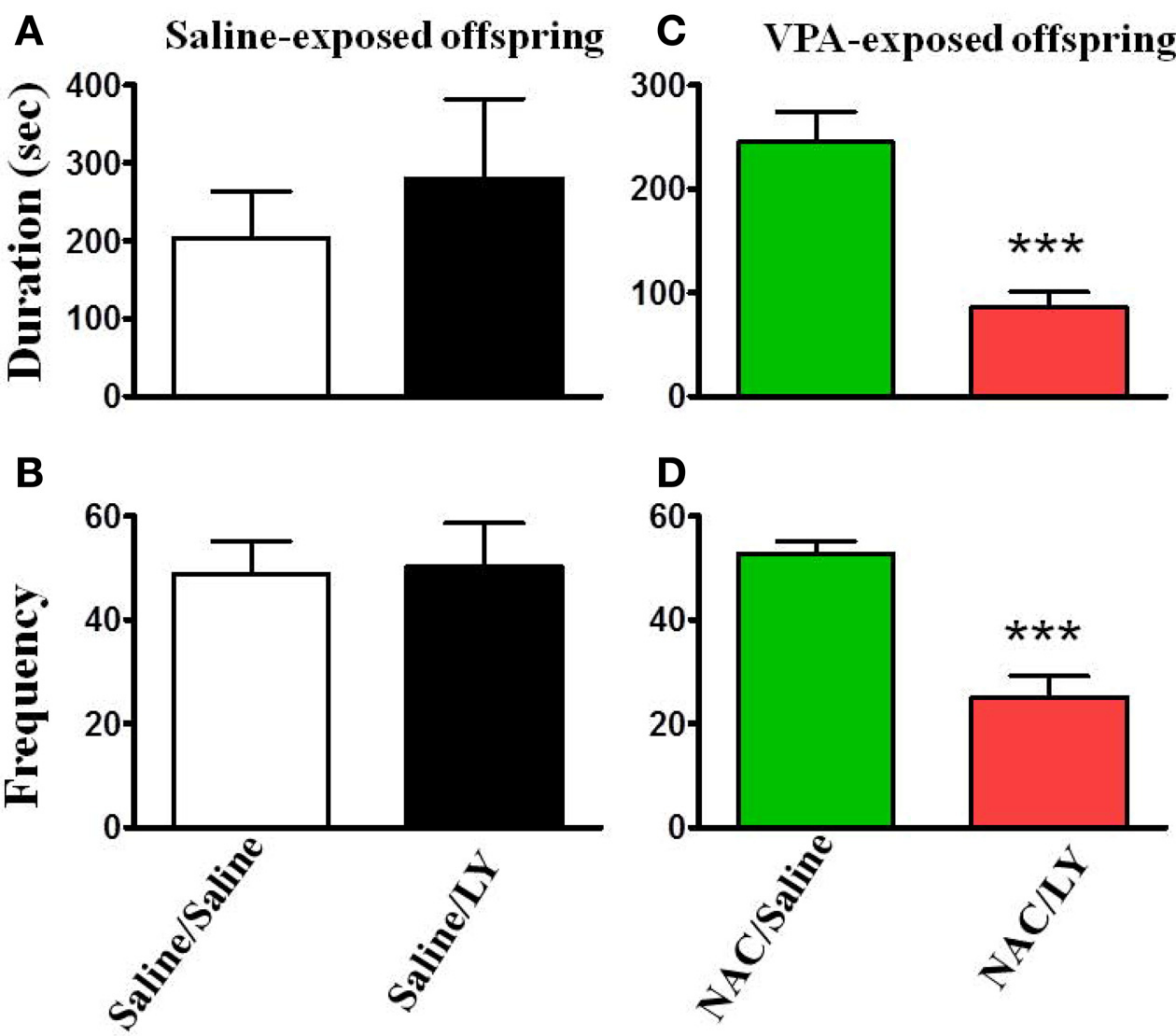
**NAC-mediated reversal of social interaction deficit in VPA-exposed offspring is blocked by mGluR2/3 antagonist. (A,B)** Rats of saline-exposed offspring weaned at postnatal day 21. At postnatal day 24, the rats received an intra-amygdala injection of LY341495 (2 μg/0.5 μl per side) or saline once per day for 5 days. One hour after the last injection of LY341495 or saline, the rats were administered the social interaction test. The duration **(A)** and frequency **(B)** of contact between the VPA- and saline-exposed offspring were measured for 20 min. Sample sizes (*n*): Saline/Saline *n* = 4, Saline/LY *n* = 5. **(C,D)** Rats of VPA-exposed offspring weaned at postnatal day 21 were administered an intraperitoneal injection of NAC (150 mg/kg) once per day for 8 days. At postnatal day 24, the rats received an intra-amygdala injection of LY341495 (2 μg/0.5 μl per side) or saline 30 min before NAC once per day for 5 days. One hour after the last injection of LY341495 or saline, the rats were administered the social interaction test. The duration **(A)** and frequency **(B)** of contact between the VPA- and saline-exposed offspring were measured for 20 min. Sample sizes (*n*): Saline/Saline *n* = 9, Saline/LY *n* = 9. ^***^p < 0.001 vs. NAC/Saline.

NAC is a cystine prodrug that increases extracellular glutamate by stimulating system Xc^−^, resulting in activation of presynaptic mGluR2/3 and thereby reducing synaptic glutamate release (Baker et al., 2002). We tested this hypothesis by infusion of the mGluR2/3 antagonist LY341495 (2 μg/0.5 μl/side) into the amygdala 30 min before the administration of NAC. Figure 6 shows that LY341495 reduced both the interaction duration (Figure 6C) and frequency (Figure 6D) to the levels of 85.4 ± 15.5 s (*n* = 9) and 32.1 ± 2.3 (*n* = 9), respectively, which were comparable to those of the VPA/saline (duration: 108.7 ± 14.9 s; frequency: 25.1 ± 4.1, *n* = 27) rats. As a control experiment, we also infused LY341495 into the amygdala in saline-exposed offspring. LY341495 had no effect on either the interaction duration (Figure 6A) or frequency (Figure 6B). These results indicated that LY341495 blocked the reversing effects of NAC on social interaction.

**Figure 7:**
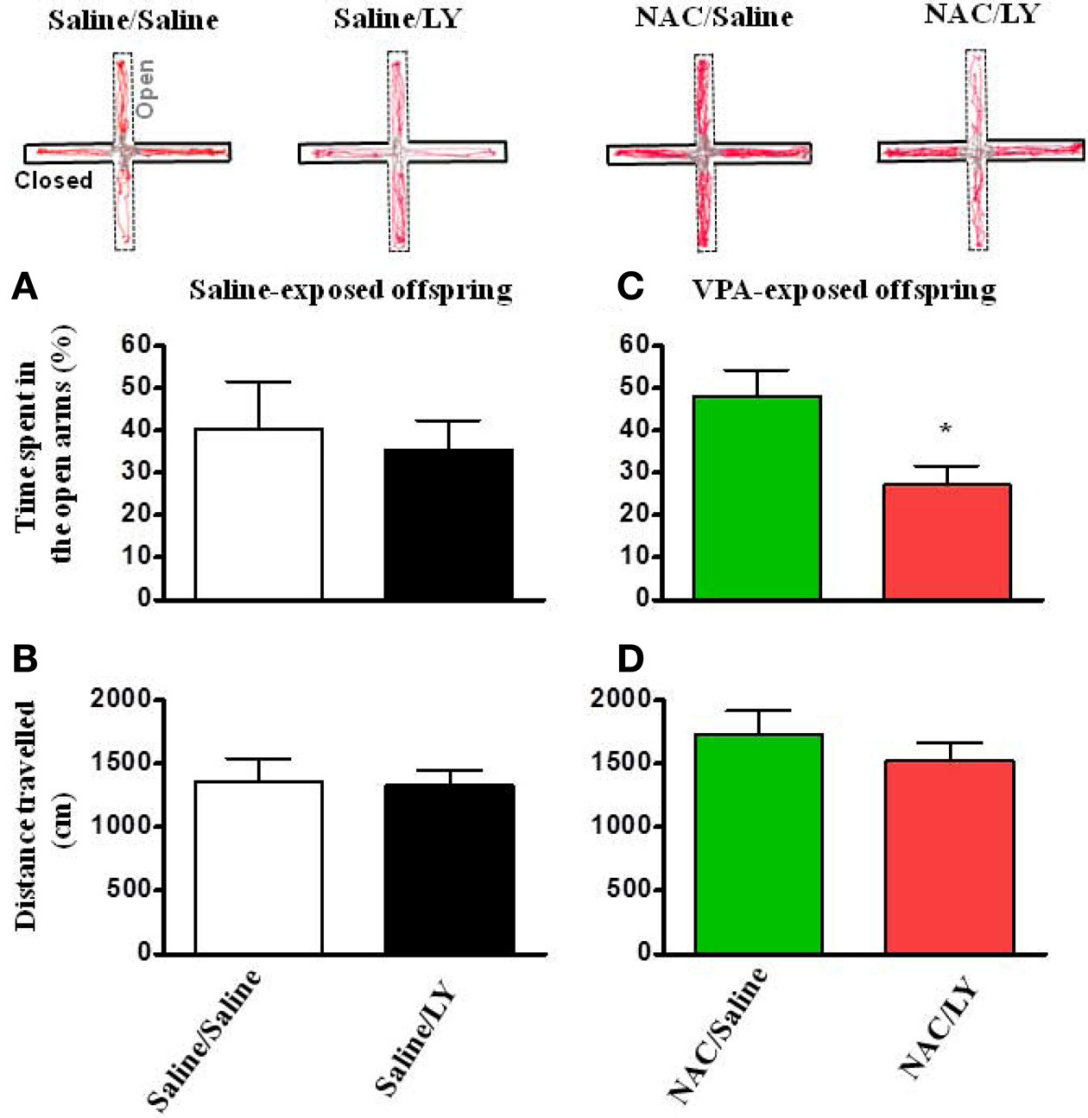
**NAC-mediated amelioration of anxiety in VPA-exposed offspring is blocked by mGluR2/3 antagonist. (A,B)** Rats of saline-exposed offspring weaned at postnatal day 24 received an intra-amygdala injection of LY341495 (2 μg/0.5 μl per side) once per day for 5 days. Twenty-four hours later, the rats were administered the elevated plus-maze test. The time spent in the open arms **(A)** and the distance traveled **(B)** were measured for 5 min. **(C,D)** Rats of VPA-exposed offspring weaned at postnatal day 21 were administered an intraperitoneal injection of NAC (150 mg/kg) once per day for 8 days. At postnatal day 24, the rats received an intra-amygdala injection of LY341495 (2 μg/0.5 μl per side) 30 min before NAC once per day for 4 days. Twenty-four hours after the last injection of NAC and LY341495, the rats were administered the elevated plus-maze test. The time spent in the open arms **(C)** and the distance traveled **(D)** were measured for 5 min. ^*^p < 0.05 vs. NAC/Saline.

Similar to the effects on social interaction, in the open field test, LY341495 also blocked the reversing effects of NAC on the time spent in the center field in VPA-exposed offspring (Figure 7C) without affecting the total distance traveled (Figures 7B,D).

### The expression of the mGluR2/3 receptor is reduced in VPA-exposed offspring

**Figure 8:**
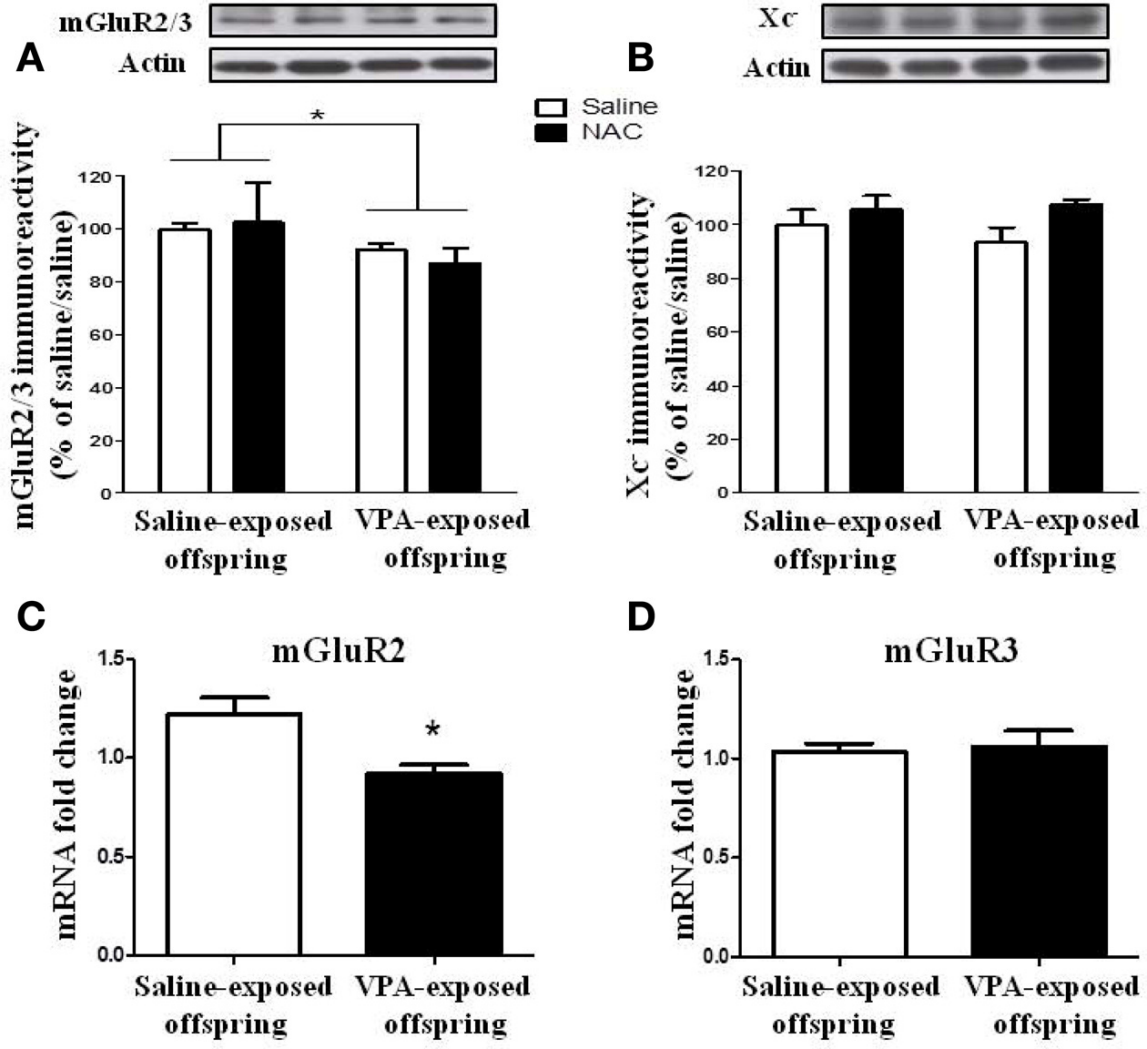
**The expression of the mGluR2/3 receptor is reduced in VPA-exposed offspring. (A,B)** Rats of saline- or VPA-exposed offspring weaned at postnatal day 21 were administered NAC (150 mg/kg, i.p.) or saline once per day for 7 days. Twenty-four hours after the last injection of NAC, the amygdala mGluR2/3 **(A)** and Xc^−^ **(B)** protein levels were determined by Western blotting analysis. ^*^p < 0.05. **(C,D)** The mRNA levels of mGluR2 and mGluR3 were determined by RT-PCR analysis.

We determined whether the expressions of the mGluR2/3 receptor and Xc^−^ were altered in the VPA-exposed offspring. Two-Way ANOVA revealed a significant main effect of pre-treatment [VPA vs. saline, *F*_(1, 20)_ = 5.493, *p* < 0.05] in the absence of a main effect of drug [NAC vs. saline, *F*_(1, 20)_ = 0.072, *p* > 0.5], or a significant pre-treatment x drug interaction [*F*_(1, 20)_ = 0.59, *p* > 0.1) (Figure 8A). These results suggested that the expression of the mGluR2/3 receptor was significantly lower in the VPA-exposed group than in the saline-exposed offspring. In addition, treatment with NAC had no effect on the expression of the mGluR2/3 receptor. In contrast to the expression of mGluR2/3, there were no significant effects of pre-treatment [VPA vs. saline, *F*_(1, 21)_ = 0.148, *p* > 0.5] or drug [NAC vs. saline, *F*_(1, 20)_ = 3.019, *p* > 0.05] and no significant pre-treatment x drug interaction [*F*_(1, 21)_ = 0.562, *p* > 0.1] in Xc^−^ expression among the four groups of rats (Figure 8B).

We also measured the *mGluR2* and *mGluR3* mRNA levels. The results revealed that the *mGluR2* mRNA level in the VPA-exposed offspring rats was significantly lower than that in the saline-exposed control rats (*p* < 0.05) (Figure 8C). In contrast, there was no difference between these two groups in terms of *mGluR3* mRNA (*p* > 0.5) (Figure 8D).

## Discussion

We have confirmed that the male VPA-exposed offspring exhibited a deficit in social interaction and increased anxiety in the EPM and open field tests. Whole-cell recordings revealed increased frequency of mEPSCs and decreased PPF at the thalamo-LA synapses from brain slices of VPA-exposed offspring. The frequency of mEPSCs is a functional index of the number of glutamate release sites per cell. PPF is generally attributed to a presynaptic change in release probability that is decreased when release of the transmitter is increased (Hsia et al., 1998; Zucker and Regehr, 2002). The observed increased frequency and increased PPF, coupled with an unaltered amplitude of mEPSCs, suggest that the presynaptic release mechanism may underlie the behavioral abnormality in this rat model of autism. The protein levels of mGluR2/3 were reduced, which was correlated with the decrease in mGluR2 mRNA. In contrast, mGluR3 mRNA was unchanged. Thus, these results suggested that mGluR2 might play an important role in the underlying mechanisms of ASD.

The mechanisms through which NAC reverses the amygdal presynaptic efficiency of excitatory synaptic transmissions remain to be explored. Previous studies have implied that NAC affects glutamate homeostasis at the nucleus accumbens synapse in cocaine seeking. NAC activated presynatic mGluR2/3 receptors-mediated depotentiation but not post-synaptic mGluR5 receptor led to reduce the EPSCs (Kalivas, 2009; Reissner and Kalivas, 2010; Kupchik et al., 2012). Interestingly, we found in this study that NAC improved social interaction and ameliorated anxiety-like behaviors in the VPA-exposed offspring. In addition, NAC treatment reversed the increased frequency of mEPSCs and decreased the PPF in the VPA-exposed offspring. All these effects could be blocked by mGluR2/3 receptors antagonist LY341495. The present study was consistent with previous study of addition disorders, and the results showed that NAC could decrease the PPF at the thalamo-LA synapses in the VPA-exposed offspring via activation of presynaptic mGluR2/3 receptors.

We also found that the protein levels of mGluR2/3 and the mRNA level of mGluR2 were decreased in VPA-exposed offspring. Hascup et al. noted that a novel positive allosteric modulator (PAM) of mGluR2 can inhibit the enhanced phasic glutamate release within the prefrontal cortex in the restraint-stressed rat. Thus, the PAM of mGluR2 has the potential effect of an anxiolytic in restraint stress (Hascup et al., 2012). Our study also noted that the regulation of mGluR2 played dual roles in VPA-exposed offspring. Future experiments to investigate the role of mGluR2 in the ASD model are warranted.

In the present study, we provided the first evidence that the abnormal behavioral performances and electrophysiological characteristics in VPA-exposed offspring could be corrected after administration of NAC. NAC is the N-acetyl derivative of the amino acid L-cysteine, which is rapidly oxidized to cystine in the pro-oxidant milieu of the brain. The cystine-glutamate antiporter (system Xc^−^) is responsible for the control of extracellular glutamate and feedback regulation of glutamate release (Baker et al., 2002). NAC administration can activate the cystine-glutamate antiporter through provision of additional cystine, thus increasing the extracellular glutamate concentration. The extracellular glutamate activates metabotropic glutamate receptors (mGluR2) on presynaptic neurons and reduces vesicular glutamate neurotransmission. Consistent with this hypothesis, we found that NAC-mediated reversal of the behavioral phenotype was blocked by mGluR2/3 antagonist.

If the reduced expression of mGluR2 underlies the abnormal behaviors seen in VPA-exposed offspring, then administration of mGluR2/3 antagonist to normal rats may cause autism-like phenotypes. However, this is not the case, as intra-amygdala infusion of mGluR2/3 antagonist LY341495 had no effect on social interaction (Figure 6A) and the results of the open field test (Figure 7A) in the saline-exposed offspring. It is likely that the half-life of LY341495 in the brain pharmacokinetics (4.75 h) (Ornstein et al., 1998) is too short to produce autism-like behaviors. Future study with genetic knockdown of mGluR2 may resolve this issue.

In summary, in a rat model of valproate-induced autism, we found that the protein and mRNA levels of mGluR2 were reduced concomitant with an increased frequency of mEPSCs and decreased PPF, which could be corrected by NAC. Thus, medications that could enhance mGluR2 expression and/or reduce glutamate release in the amygdala might be candidates for the treatment of ASD.

## Author contributions

Corresponding author Po See Chen designed the study and wrote the protocol. Authors Po-Wu Gean and Chao-Chuan Wang helped to design the study. Authors Ming-Chong Ng and Ya-Hsin Hsiao contributed to the statistical analyses. Authors Yu-Wen Chen and Hui-Ching Lin wrote the first draft of the manuscript. Authors Ming-Chong Ng and Yu-Wen Chen managed the data collection. All authors interpreted the analysis of the results and helped to revise the manuscript. All authors approve the final version for publication and agree to be accountable for all aspects of the work in ensuring that questions related to the accuracy or integrity of any part of the work are appropriately investigated and resolved.

### Conflict of interest statement

The authors declare that the research was conducted in the absence of any commercial or financial relationships that could be construed as a potential conflict of interest.
